# Non-coding RNAs as Regulators of Cellular Senescence in Idiopathic Pulmonary Fibrosis and Chronic Obstructive Pulmonary Disease

**DOI:** 10.3389/fmed.2020.603047

**Published:** 2020-12-23

**Authors:** Norihito Omote, Maor Sauler

**Affiliations:** Pulmonary, Critical Care and Sleep Medicine Section, Department of Internal Medicine, Yale University School of Medicine, New Haven, CT, United States

**Keywords:** COPD–Chronic Obstructive Pulmonary Disease, IPF–Idiopathic Pulmonary Fibrosis, cellular senescence, non-coding RNA, micro-RNA (miRNA), long-non coding RNA

## Abstract

Cellular senescence is a cell fate implicated in the pathogenesis of idiopathic pulmonary fibrosis (IPF) and chronic obstructive pulmonary disease (COPD). Cellular senescence occurs in response to cellular stressors such as oxidative stress, DNA damage, telomere shortening, and mitochondrial dysfunction. Whether these stresses induce cellular senescence or an alternative cell fate depends on the type and magnitude of cellular stress, but also on intrinsic factors regulating the cellular stress response. Non-coding RNAs, including both microRNAs and long non-coding RNAs, are key regulators of cellular stress responses and susceptibility to cellular senescence. In this review, we will discuss cellular mechanisms that contribute to senescence in IPF and COPD and highlight recent advances in our understanding of how these processes are influenced by non-coding RNAs. We will also discuss the potential therapeutic role for targeting non-coding RNAs to treat these chronic lung diseases.

## Introduction

Chronic obstructive pulmonary disease (COPD) and idiopathic pulmonary fibrosis (IPF) are chronic lung diseases that disproportionately affect the elderly and impose a significant global health burden. IPF is characterized by chronic and progressive lung scarring while COPD is characterized by heterogenous manifestations of emphysema, small airway disease, and chronic bronchitis. Despite their distinct pathologic features, both diseases are epidemiologically and biologically associated with aging (1–3). The prevalence of COPD amongst individuals aged ≥75 years is approximately 10% compared with 3–4% in those 25–44 years (1, 4) and the prevalence of IPF amongst individuals aged ≥75 years is 0.2–0.3% compared to 0.004–0.012% cases in those aged 35–44 years (5). Additionally, the pathogenesis of IPF and COPD involve biologic “hallmarks of aging” (1, 2, 6–10). These “hallmarks of aging,” first described by Lopez-Otín et al., are cellular processes that occur more frequently with age, contribute to aging-related functional decline, and can be experimentally manipulated to accelerate or slow aging in model organisms (11). One biologic “hallmark of aging” that has emerged as a therapeutic target for ILD, COPD, and other age-related disorders is cellular senescence.

Cellular senescence is a cell fate that occurs in response to diverse causes of cellular stress, such as DNA damage, oxidative stress, telomere shortening, and oncogene activation (12, 13). Cellular senescence is characterized by permanent cell cycle arrest due to persistent activation of p16^INK4a^-RB (retinoblastoma) and p53-p21^CIP1/WAF1^ pathways (14, 15). However, senescent cells frequently have altered cellular metabolisms, reorganized chromatin, and activated damage sensing pathways (e.g., p38 MAPK and NF-κB), and are apoptosis resistant. They also adopt a senescence associated secretory phenotype (SASP) and secrete high levels of cytokines, chemokines, and matrix metallopeptidases (MMPs). The biologic consequences of cellular senescence are complex because senescence has both beneficial and detrimental effects. Cellular senescence is critical for embryogenesis, promotes wound healing, and mitigates malignant transformation. However, the accumulation of senescent cells with age also causes chronic inflammation, extracellular matrix alterations, a decline in tissue regeneration, and an increased risk for many aging-related disorders (16).

Cellular senescence is just one of many potential cell fates. Cells maintain diverse stress responses that can resolve cellular stress or activate alternative cell fate pathways such as programmed cell death (e.g., apoptosis/necroptosis), quiescence, or differentiation. While cell fate is influenced by the type, magnitude, and duration of cellular stress, microenvironmental and intracellular factors also influence cell fate “decisions” through modulation of intracellular signaling networks. Consequently, susceptibility to cellular senescence varies across cell/tissue types and with age and in disease (17). There is increasing recognition for the important role of non-coding RNAs in the regulation of signaling networks that influence susceptibility to cellular senescence (18, 19). In this review, we will highlight non-coding RNAs that regulate senescence-associated molecular pathways in the context of IPF and COPD pathogenesis and discuss current approaches and challenges for therapeutically targeting non-coding RNAs for these diseases.

## Cellular Senescence and Its Causes in the Pathogenesis of IPF and COPD

Cellular senescence is now considered an important mechanism of IPF and COPD pathogenesis (20, 21). In IPF, cellular senescence markers are increased in epithelial and mesenchymal cells within remodeled areas of fibrotic lung, and eliminating senescent cells using genetically modified mice or pharmacologic agents decreases disease severity in animal models of pulmonary fibrosis (22–26). Singe-cell RNA sequencing studies suggest epithelial senescence in IPF occurs in a unique subpopulation of cells that reside adjacent to myofibroblasts and may arise from the persistence of a transitional alveolar epithelial cell state (27, 28). These cells express p16, p21, certain basal cell markers, developmental and epithelial-mesenchymal transition markers, and may be a source of pro-fibrotic SASP signaling (29, 30). Additionally, while fibroblast senescence is important for normal wound healing, IPF pathogenesis may involve the persistence of senescent fibroblasts that secrete pro-fibrotic mediators and senescent myofibroblasts that are apoptosis resistant (23, 31). Lungs of patients with COPD demonstrate increased markers of cellular senescence in epithelial cells, fibroblasts, and endothelial cells and many (albeit not all) studies have demonstrated genetic or pharmacologic inhibition of cellular senescence can mitigate disease severity in animal models of COPD (32–37). It has also been postulated that chronic inflammation and airway remodeling in COPD may arise from the production of pro-inflammatory SASP factors (38). Additionally, impaired tissue repair may be the result of reduced replicative capacity in senescent progenitor cells.

Cellular senescence in IPF and COPD is commonly caused by oxidative stress and DNA damage (39). Oxidative stress refers to an imbalance between reactive oxygen/nitrogen species (ROS/RNS) and cellular antioxidants. While ROS can arise from many exogenous sources (e.g., cigarette smoke) and chronic inflammation, one of the most abundant sources of ROS are mitochondria. There is increased mitochondrial ROS production and mitochondrial dysfunction with age and in IPF and COPD (40–42). In addition, IPF and COPD are associated with increased oxidative biomarkers, and consequences of oxidative stress including macromolecular damage (e.g., protein, DNA, and organelle damage), inflammation, cellular senescence and cell death (26, 32, 43). DNA damage is another important cause of cellular senescence, through activation of p53, increased p21 and p16 transcription, and stabilization of GATA4 (12, 15, 44). DNA damage is increased in IPF and COPD, and inadequate DNA repair capacity may contribute to disease progression in COPD (45–47). Telomere shortening can also activate DNA damage responses (17, 48). Normally, a shelterin complex protects telomeric strands from being recognized by DNA damage responses. However, telomere shortening can cause loss of the shelterin complex, telomere “uncapping,” DNA damage response activation, and cellular senescence. In IPF, shortened telomeres and mutations in telomere maintenance genes are well-described risk factors for disease (49–51). Similarly, telomerase mutations are risk factors for early onset emphysema and telomere length is associated with severity of airflow limitation, increased risk for acute exacerbations, and increased mortality (34, 52–55).

To combat cellular stress, cells maintain a repertoire of cellular stress responses, but many of these adaptive responses wane with age or are decreased in IPF and COPD. For example, NRF2 is a transcription factor that promotes the production of cellular antioxidants and detoxifying enzymes. However, NRF2 activity decreases with age and impaired NRF2 activity is implicated in the pathogenesis of IPF and COPD (31, 56–59). Another adaptive stress response is autophagy, a process in which cells degrade and “recycle” damaged proteins and organelles through lysosome-dependent pathways. With age, there is reduced autophagy and mitophagy, a selective type of autophagy for the specific degradation of mitochondria. Consequently, there is a reduced capacity to alleviate consequences of oxidative stress and increased susceptibility to cellular senescence (60, 61). In IPF, reduced autophagy in epithelial cells and fibroblasts increase susceptibility to cellular senescence and disease pathogenesis (62–66). Similarly, deficient mitophagy and its mediators, including PINK1 and SIRT3, impair mitochondrial function, increase mitochondrial ROS production, and contribute to progressive fibrosis in IPF (9, 10, 67). While autophagy is an adaptive response, persistent autophagy can cause activation of cell death and cell senescence pathways as well (68, 69). Autophagy and mitophagy are increased in severe COPD, and both insufficient and excess autophagy and mitophagy are implicated in COPD pathogenesis (70–74).

Collectively, these findings underscore the increasing evidence that the pathogeneses of IPF and COPD involve cellular senescence and dysregulation of stress responses that mitigate cellular senescence. Therefore, regulatory factors that increase or reduce susceptibility to cellular senescence may represent novel therapeutic targets for these diseases.

## Non-Coding RNAs in Aging, IPF, AND COPD

Non-coding RNAs lack protein coding capacity but still regulate diverse cellular processes including those implicated in aging biology, cellular senescence, and the pathogeneses of IPF and COPD. Non-coding RNAs are mainly classified into two groups, microRNAs (miRNAs) and long non-coding RNAs (lncRNAs). miRNAs are small 18–25 base single stranded RNA molecules (75). They are initially transcribed as primary-miRNA (pri-miRNA) molecules that fold into a stem loop structure. Subsequently these pri-miRNAs undergo sequential processing by enzymes Drosha and Dicer to generate miRNA strand duplexes. The mature miRNA strand of the duplex is then loaded into a miRNA-induced silencing complex (miRISC) where it binds complementary mRNA sequence to inhibit mRNA translation or promote mRNA degradation. Typically, miRNAs bind the 3′ untranslated region (UTR) of mRNA but can bind other regions as well. Because one single miRNA can target hundreds of mRNAs, miRNAs can modulate complex biologic processes including those related to lifespan and aging (76, 77). In humans, age-related changes in miRNA expression have been identified in lung, peripheral blood mononuclear cells (PBMCs) and serum (18, 78–81).

LncRNAs are a diverse group of non-coding RNAs longer than 200 nucleotides (82, 83). Certain lncRNAs are transcribed from intergenic regions (long intergenic non-coding RNAs or lincRNAs), while others are derived from excised introns. Sense lncRNAs are located in proximity to a coding gene on the sense strand while antisense lncRNAs are transcribed from the opposite strand of a coding gene. Certain lncRNAs undergo capping, splicing, and polyadenylation much like mRNAs, while others undergo alternative post-transcriptional processing, such as forming circular molecules or processing by RNase P to form stabilizing triple helix structures at their 3′ ends (84). LncRNAs are also functionally diverse. They can act as cis- or trans- regulatory elements to enhance or inhibit mRNA transcription and/or mRNA translation. LncRNAs can mediate their regulator effects by affecting chromosomal architecture, modulating the recruitment of chromatin modifiers, binding DNA directly to form complex structures that interfere with transcriptional machinery, or binding complementary mRNA transcripts to regulate their stability, splicing, or post-transcriptional modification (84–86). Similar to microRNAs, lncRNAs have also been implicated in aging biology (87, 88).

Non-coding RNAs are implicated in the pathogeneses of IPF and COPD and are emerging targets for therapeutic intervention. Approximately 10% of miRNAs are significantly changed in IPF lungs, including a decrease in miR-29, miR-30, let-7, miR-96, and miR-17–92 family members and an increase in miR-154, miR-155, miR-34, miR-26, miR-200 and miR-21 family members (89–91). miRNA expression can be altered by cigarette smoke or by the presence of COPD (92, 93). Studies profiling miRNAs in COPD lung tissue samples have demonstrated increased expression of miR-34a, miR-146a, miR-144, miR-15b, miR-570, and decreased expression of miR-24 and mir-218 (94–99). Other studies evaluating the expression of miRNAs in serum and sputum samples have found differential expression of miR-21, let-7c, miR-610, miR-34 a/b/c, let-7c, miR-146a, miR-125b, and miR-199a with COPD (93, 95, 97, 100–102). The differentially expressed lncRNAs in IPF or animal models of lung fibrosis include MEG3, TERRA, SIRT1-AS, MALAT1, FENDRR, and DNM3OS (103–108). Studies of lncRNAs in COPD lung tissue have identified differential expression of MEG3, ANRIL, SAL-RNA, and SCAL1 with COPD (97, 109, 110). Many of these differentially expressed non-coding RNAs in IPF and COPD have been shown to regulate various aspects of aging biology and cellular senescence. Below, we provide examples of such non-coding RNAs and discuss how their regulation of aging biology and cellular senescence may contribute to disease pathogenesis (Tables 1, 2).

**Table 1:** Aging related non-coding RNAs in IPF.

**Non-coding RNAs**		**Expression in IPF**	***In vivo* role in fibrosis**	**Senescence related mechanisms**
microRNAs	mir-34a	↑Lung	Antifibrotic (young)	Inhibits cellular senescence through SIRT1 in AECs and fibroblasts
			Profibrotic (aged)	
	mir-29	↓Lung	Antifibrotic	Increases AEC antioxidants (SOD2, MnSOD, catalase) Inhibits apoptosis in AECs by regulating FOXO3A
	mir-17~92	↓Lung	Antifibrotic	Inhibits mTOR, promotes autophagy, decreases cellular senescence
	mir-200 family	↓Lung	Antifibrotic	Inhibits AEC cellular senescence and epithelial mesenchymal transition
lncRNAs	SIRT1-AS	↓Lung	–	Inhibits miR-34a-mediated targeting of SIRT1
	TERRA	↑Lung	–	Promotes telomere maintenance
	MALAT1	↓Lung	Antifibrotic	Stabilizes the antioxidant NRF2
	LincRNA-p21	↑Lung	–	Promotes cellular senescence through activation of p53 and p21

*AEC, alveolar epithelial cells*.

**Table 2:** Aging related non-coding RNAs in COPD.

**Non-coding RNAs**		**Expression in COPD**	***In vivo* role in COPD**	**Senescence related mechanisms**
microRNAs	mir-34a	↑Lung	Increases susceptibility to emphysema	Promotes cellular senescence by inhibition of SIRT1/6
		↓BAL		
	mir-570	–	–	Promotes cellular senescence by inhibition of SIRT1
				
	mir-24	↓Lung	Protects against emphysema	Inhibits DNA repair and apoptosis
	mir-126	↓Blood outgrowth endothelial cells	–	Inhibits DNA damage response
	mir-218	↓Lung and Sputum	Protects against cigarette smoke induced inflammation	Inhibits cellular senescence via BMI1
lncRNAs	ANRIL	↓Plasma	–	Inhibits p16 expression and SASP cytokine production
	(CDKN2B-AS1)			
	SCAL1	↑Airway epithelium	–	Activates antioxidant responses downstream of NRF2
	MEG3	↑Lung	–	Promotes p53 activity

### Non-coding RNAs in COPD and IPF

#### miR-34 and miR-570 Regulation of Sirtuins in IPF and COPD

The miR-34 family consists of three members: miR-34a, miR-34b, and miR-34c. They are direct transcriptional target of p53 and therefore can be induced by oxidative and genotoxic stress (111). Members of the miR-34 family are encoded by two different genes; miR-34a is encoded within chromosome 1 whereas miR-34b and miR-34c are encoded within chromosome 11. Studies show miR-34a expression increases with age and can promote cellular senescence in part through negative regulation sirtuins, particularly SIRT1 and SIRT6 (111–113). Sirtuins are nicotinamide adenine dinucleotide (NAD)-dependent molecules that promote longevity by regulating diverse cellular processes including: cellular senescence, inflammation, DNA repair, autophagy, mitochondrial generation and mitochondrial ROS production (114, 115). For example, SIRT1 can function as a histone deacetylase to negatively regulate NF-κB, mitochondrial biogenesis, p53, p21, and p16 (116, 117). In the lung, miR-34a is expressed in type II alveolar epithelial cells (AECs) and fibroblasts, and increased miR-34a expression coupled with reduced SIRT1 and SIRT6 expression are associated with IPF and COPD (118–123).

Previous studies demonstrate miR-34a is increased in type II AECs from patients with IPF and in murine models of lung fibrosis (124, 125). Both *in vitro* and *in vivo* experiments demonstrate miR-34a promotes cellular senescence in part through inactivation of SIRT1 and increased mitochondrial dysfunction (118, 119, 124). Interestingly, the consequences of miR-34a genetic deletion in mice are age-dependent. miR-34a protects against lung fibrosis by increasing fibroblast susceptibility to cellular senescence in young mice, while miR-34a promotes lung fibrosis by increasing alveolar epithelial susceptibility to cellular senescence and apoptosis in old mice (119, 124). The divergent roles for miR-34a in young and old mice underscore the complex temporal- and cell type-specific roles for cellular senescence in disease pathogenesis.

miR-34a is also increased COPD lungs. In airway epithelial cells and lung tissue, miR-34a expression is induced by oxidative stress and inversely correlates with SIRT1 and SIRT6 expression (99, 126). In a murine model of COPD, miR-34a inhibitors increase SIRT1 and SIRT6 expression and reduce NF-κB signaling, matrix metalloproteinase expression, cellular senescence, and emphysema severity. (121–123, 126). Another sirtuin regulator in COPD is miR-570, which is located at chromosome 3 and targets the 3′-UTR of SIRT1 mRNA for degradation. miR-570 expression is induced by oxidative stress and increased in lung tissue and airway epithelial cells from patients with COPD (98). Inhibition of miR-570 reduces cellular senescence and the secretion of SASP factors such as IL-6, IL-1, and CXCL8. Together, these data demonstrate the important roles of miR-34a and miR-570 in regulation of cellular senescence and susceptibility to IPF and COPD through modulation of sirtuins.

#### miR-29 and IPF

The roles of miR-29 family members in IPF are context dependent and underscore the complex interactions of microRNAs, aging biology, and disease pathogenesis. There are three mature members of the miR-29 family, miR-29a, miR-29b, and miR-29c, which are encoded within two bicistronic clusters (miR-29a/miR-29b-1 located on chromosome 7 and miR-29b-2/mir-29c located on chromosomes 1) (127, 128). In the lung, miR-29 is largely expressed in mesenchymal and epithelial cells where its expression is associated with oxidative stress, DNA damage, and cellular senescence (129–131). However, miR-29c is decreased in IPF lung tissue samples and experimentally induced fibrosis in mouse lungs (124, 132). miR-29c deficiency in type II AECs increases susceptibility to apoptosis and reduces their capacity for epithelial renewal while miR-29c mimics protect type II AECs from apoptosis by regulating FOXO3A and increasing expression of ROS-neutralizing enzymes such as SOD2, MnSOD and catalase (133). miR-29b mimics can inhibit bleomycin-induced lung fibrosis, fibroblast production of extracellular matrix, expression of IGF-1 and production of inflammatory cytokines such as IL-4 and IL-12 (128, 134). Therefore, an increase in miR-29 with oxidative stress, cellular senescence, or with age may be an endogenous response that protects against fibrosis, and a loss of this adaptive response may contribute to the pathogenesis of IPF.

#### miR-17~92 Cluster and miR-200 Family in IPF

Both the miR*-17*~*92* cluster and miR-200 family regulate susceptibility to cellular senescence in IPF. The miR*-17*~*92* cluster encodes 6 miRNAs (miR-17, miR-18a, miR-19a, miR-20a, miR-19b-1, and miR-92a) on chromosome 13 and is frequently decreased in multiple tissue types with age and in senescent cells (135). miR-17~92 decreases susceptibility to cellular senescence through diverse mechanisms including targeting cell cycle proteins, inhibition of the mechanistic target of rapamycin (mTOR), and activation of autophagy (136). Members of the miR-17~92 cluster are hypermethylated in lung tissue samples and fibroblasts from IPF patients, and the use of epigenetic methylation inhibitors to promote expression of the miR-17~92 cluster attenuates fibrosis in bleomycin-murine models (90). Similarly, mice overexpressing miR-17 have highly proliferative, albeit poorly differentiated, epithelial cells and decreased number of senescent cells in their lung (137, 138).

The miR-200 family consists of five members within two clusters, miRs-200a/b/429 on chromosome 1 and miRs-200c/141 on chromosome 12. These microRNAs can regulate oxidative stress, DNA repair, and cellular senescence, although the direction of effect can be context dependent (139, 140). Levels of miR-200a and miR-200c are significantly decreased in IPF lungs and in the lungs of mice with experimental lung fibrosis (141). Transfection of AECs with miR-200a and miR-141 reduces epithelial mesenchymal transition (EMT) and the expression of cellular senescence markers including p16 and p21, but does not improve AEC proliferation capacity. In contrast, transfection with miR-200b/c increases differentiation of senescent type II AECs into type I AECs, decreases EMT, and reduces disease severity in animal models of pulmonary fibrosis (142–145).

#### miR-24 and miR-126 Regulate DNA Damage Responses in COPD

miR-24 is a member of a poly-cistronic miR-23~27~24 miRNA clusters that occur in two genomic loci in humans. The miR-23b-27b-24-2 cluster is located in an intronic region of chromosome 9 while the miR-23a~27a~24-1 cluster is located in an intergenic region of chromosome 19 (146, 147). Dysregulation of miR-23~27~24 signaling has been identified in multiple age-related disorders including diabetes and Alzheimer's disease, and both oxidative and genotoxic stress have been shown to modulate expression of these miRNAs, although the direction of effect is context dependent (148, 149). In COPD, miR-24 expression inversely correlates with COPD disease severity as measured by FEV_1_ percent predicted and radiographic emphysema (150). miR-27a and miR-23a expression also inversely correlates with disease severity, albeit it to a lesser degree that miR-24. In a mouse model, inhibition of miR-24 increases susceptibility to cigarette smoke-induced emphysema. Others have found that inhibition miR-24-27-23 cluster in T-cells increases allergic airway inflammation and goblet metaplasia (151). miR-24 can inhibit the expression of p16 by targeting its 3′ UTR to inhibit cellular senescence (152). However, miR-24 can also inhibit DNA repair and the translation of DNA repair genes including H2AX, TOP1, and BRCA1, which can promote cellular senescence in certain contexts (148). Interestingly, another miRNA that inhibits DNA damage responses and is decreased in COPD is miR-126 (153–155). These collective findings suggest that microRNA inhibition of DNA damage responses may protect against COPD pathogenesis, although whether this occurs by changing susceptibility to cellular senescence remains to be determined.

#### miR-218 and COPD

The mature form of miR-218 can be transcribed from intronic regions of SLIT2 and SLIT3 located on chromosomes 4 and 5, respectively (156). miR-218 is decreased in bronchial epithelial cells of smokers and in lungs and sputum from COPD patients (93, 96). In a murine model of COPD, inhibition of miR-218 increases susceptibility to emphysema and airway inflammation with increased production of IL-8 and CCL2 (96). Notably, one of the downstream targets of miR-218 is BMI-1, a polycomb repressive group protein which inhibits p16 expression and cellular senescence (157). This raises the possibility that decreased miR-218 expression promotes cellular senescence and disease progression in COPD, although further studies are warranted.

### LncRNA

#### Lnc ANRIL (CDKN2B-AS1) and Lnc SIRT1-AS

ANRIL is transcribed from the antisense strand of CDKN2A/2B, the genes that encode cyclin-dependent kinase inhibitors p15 and p16, on chromosome 9 (158). ANRIL mediates transcriptional repression of these antisense genes through RNA-RNA interactions, as well as histone methylation and chromatin remodeling of polycomb repressive complexes (PRC) (158, 159). ANRIL activity is highly variable and dependent on tissue type. There are 21 ANRIL splice variants, including linear and circular isoforms, and ANRIL activity is highly influenced by methylation activity in its promoter region (158). In addition to its role in regulating p15 and p16, ANRIL suppresses NF-κB and can inhibit chronic inflammation (160). In one study, ANRIL expression in plasma was decreased during acute exacerbations of COPD and ANRIL expression negatively correlated with SASP related cytokines such as TNF-α, IL-1β, IL-8 and LTB-4 in stable COPD patients (161). LncRNA SIRT1 antisense (SIRT1-AS), is transcribed from the antisense strand of SIRT1 and can form RNA hybrid double strands with SIRT1 mRNA to increase its stability (162). SIRT-AS protects SIRT1 mRNA degradation by inhibiting miR-34a binding to the 3′UTR of SIRT1 (162). In one study of bleomycin-induced lung fibrosis, SIRT1-AS overexpression inhibited TGF-β-mediated EMT (163). Despite these data, more studies will be necessary to confirm the roles of ANRIL and SIRT1-AS in COPD and pulmonary fibrosis.

#### TERRA (Telomere Repeat-Containing RNA)

TERRAs are important for telomere maintenance and characterized by 5′-(UUAGGG)-3′ repeats (164, 165). These lncRNAs are commonly transcribed from the subtelomeric 20q locus in humans in response to cellular stress and telomere shortening, the later as a consequence of reduced methylation marks and loss of telomeric heterochromatin (166). TERRAs are recruited to telomeres where they form DNA-RNA hybrid R-loops. This R-loop formation regulates telomere maintenance through interactions with chromatin modifiers, telomerase, and promoting DNA repair (166, 167). TERRAs also facilitate telomere replication and promote the assembly of shelterin proteins (168, 169). However, TERRA expression is increased in the PBMCs from IPF patients and inversely correlated with the percentage of predicted force vital capacity (106). While not well-defined, TERRAs may have an important role in IPF pathogenesis.

#### MALAT1 (Metastasis Associated in Lung Adenocarcinoma Transcript-1) and SCAL1 (Cancer-Associated lncRNA-1)

Both MALAT1 and SCAL1 are lncRNAs that regulate cellular responses to oxidative stress and cellular senescence. MALAT1 is an 8.7kbp lncRNA transcribed from human chromosome 11 and is ubiquitously express in almost all human tissue (170). MALAT1 is frequently found in nuclear “speckles” and can interact with pre-mRNA splicing factors to modulate alternative mRNA splicing (171, 172). Consequently, MALAT1 can regulate the expression of cell cycle genes and can also stabilize NRF2 to attenuate oxidative stress and DNA damage (173). MALAT1 is decreased in senescent cells and in bleomycin-induced murine fibrosis where myeloid deletion of MALAT1 increases susceptibility to fibrosis and the number of profibrotic M2 macrophages (19, 108). SCAL1, a lncRNA located on chromosome 5, can be induced by oxidative stress through NRF2-mediated transcriptional activity and is increased in the airway epithelium of smokers compared to nonsmokers (97, 174). Inhibition of SCAL1 in airway epithelial cells augments cytotoxicity induced by cigarette smoke extract in vitro, suggesting SCAL1 may act downstream of NRF2 to mediate protective antioxidant responses.

#### LincRNA-p21 (Long Intergenic Non-coding RNA p21) and MEG3 (Maternally Expressed Gene 3)

Both lincRNA-21 and MEG3 are downstream targets of p53 and mediate many p53-dependent transcriptional responses. LincRNA-p21 is a transcriptional target of p53 located approximately 15 kb upstream from CDKN1A (175). LincRNA-p21 functions as a repressor of p53-dependent transcription by binding to hnRNP-K (heterogeneous nuclear ribonucleoprotein K) and interacting with PRC1 and PRC2, although these same interactions also promote p53 activity at the p21 promoter to increase p21 transcription (176, 177). In one study, lincRNA-p21 inhibited fibroblast collagen expression through downregulation of THY1 expression (178). Maternally expressed gene 3 (MEG3) is a maternally imprinted gene located on chromosome 14, and increases with age in human lung tissue and PBMCs due to changes in promoter methylation (87, 179). Like lincRNA-21, MEG3 also promotes p53 activity. MEG3 interactions with p53 inhibit p53 ubiquitination and MDM2-mediated degradation. MEG3 can also selectively upregulate certain p53 target genes, such as GDF15, and interact with PRC1/2 to mediate p53-dependent gene silencing (180–182). Intriguingly, there are 27 known splice variants of MEG3, and changes in the relative abundance of these slice variants in response to cellular stress can modulate p53 activity (183). MEG3 is increased in the lungs of patients with COPD (184, 185). Additionally, epithelial MEG3 expression has been shown to be induced by cigarette smoke, correlate with disease severity, and promote inflammation and apoptosis through a mechanism involving miR-218 (186). MEG3 expression is also increased in atypical IPF epithelial cells and can impair basal cell differentiation, which may contribute to abnormal tissue remodeling (105). Notably, p53 can induce both cellular senescence and apoptosis in a context-dependent manner, but the role of lincRNA-p21 and MEG3 in regulating p53-mediated cell fate responses in the lung remain unknown. Additionally, while p53 is implicated in the pathogenesis of IPF and COPD, more studies are necessary to determine the roles of lincRNA-p21 and MEG3 in these diseases (187).

#### Therapeutic Targeting of Non-coding RNAs

There is a growing interest in targeting non-coding RNAs to treat chronic lung diseases due to their regulatory functions and roles in disease pathogenesis (85, 188). Therapeutic approaches for RNA targeting utilize nucleotides with complementary sequences to prevent RNA transcription, promote RNA degradation, or interfere with post-transcriptional processing of target RNAs. Catalytically dead RNA-guided CAS9 endonucleases that target specific DNA sequences can be used to hinder RNA transcription. Single-stranded antisense oligonucleotides (ASOs) that bind RNA molecules through complementary sequences promote RNA degradation through RNAase-H dependent cleavage, although newer ASOs inhibit mRNA translation through steric hindrance or interfering with normal mRNA splicing. Similarly, double-stranded RNA molecules, including small interfering RNA (siRNA) or miRNA mimics, utilize the RISC complex to inhibit transcription or promote RNA degradation.

Nucleotide-based approaches for targeting non-coding RNAs are attractive for a variety of reasons. Many non-coding RNAs, particularly lncRNAs, are expressed in a tissue- or cell- specific manner (189). Therefore, augmenting or inhibiting their expression in a cell- or tissue- specific manner can reduce off-target effects and increase the therapeutic window. Additionally, generating oligonucleotide sequences complementary to their target sequence is a much easier task using currently available technologies than identifying small-molecule inhibitors or antibodies that can specifically target proteins of interest. Even if targeted antibodies or small molecules are identified, they commonly reduce rather than augment target molecule activity. In contrast, oligonucleotide therapies can increase the concentration of target molecule production through inhibition of negative regulators such as miRNAs. Finally, many therapeutic targets, while pathologic in certain contexts, also have important homeostatic functions. For example, oxidative stress is deleterious, but ROS are critical intracellular signaling molecules. Similarly, cellular senescence promotes aging related disorders but also prevents malignant transformation. Rather than inhibiting such integral pathways completely, a more effective therapeutic strategy may be to focus on modulating these pathways by targeting regulatory non-coding RNAs.

However, nucleic acid-based therapies are not without challenges (190). First, oligonucleotides are susceptible to degradation by extracellular and intracellular nucleases. To overcome this challenge and increase oligonucleotide stability, researchers have used chemically modified phosphate backbones. For example, antagomirs are ASOs that commonly contain 2′-O-methyl or phosphonothioate modifications to improve stability. Locked nucleic acids are another commonly used ASO that utilizes a modified RNA-DNA-RNA backbone to increase binding affinity and improve stability. Certain oligoribonucleotides possess targeting moieties that can deliver nucleic acid-based therapies to specific tissue. Another challenge is that nucleotides are large negatively charged molecules and therefore do not easily cross the cell membrane. Therefore, lipid-, peptide-, and polymer-based nanoparticles have been used to deliver oligonucleotides to the cytosol. Some of these nanoparticles promote the specific uptake of oligonucleotides into the lung or increase retention within the lung following inhalation. (191, 192). Nucleic acid-based therapies are capable of promoting inflammation through toll-like receptors and other innate immune receptors for foreign DNA and RNA, although this problem can be mitigated through assays to test for immune activation and reducing CpG elements (85). Finally, non-coding RNAs can target hundreds of genes and/or function through diverse mechanisms, and therefore targeting non-coding RNAs may cause unwanted effects.

Several oligonucleotide therapies that target mRNAs have already been approved by the U.S. Food and Drug Administration for treating disease, and there are currently multiple clinical trials targeting non-coding RNAs. For example, Remlarsen, a first-generation miR-29 mimic, is currently being evaluated in a Phase 2 clinical trial assessing its safety and efficacy in skin fibrosis (193). MRX-34 is a liposomal nanoparticle formulation of a miR-34 mimic that was under investigation in a Phase I trial for cancer, but the study was stopped short because of serious adverse events (194). While lncRNAs have not been tested in clinical trials, therapeutic manipulation of MALAT1 and MEG3 have shown benefit in preclinical transgenic and xenograft models of cancer (195–197). Lin et al. reported that the knockdown of lncRNA MALAT1 via tail injection of RNAi can improve septic lung injury in mice (198). Additionally, intraperitoneal administration of ASO targeting lncRNA DNM3OS, a regulator of the TGF-β pathway, attenuated bleomycin-induced lung fibrosis in mice (104). These pre-clinical and clinical studies suggest the possibility that non-coding RNAs may have a potential therapeutic role for treating lung diseases such as IPF and COPD.

## Conclusion

Diverse cellular processes implicated in aging biology, including cellular senescence, contribute to the pathogenesis of IPF and COPD. In these diseases, cellular senescence can occur from oxidative stress, DNA damage, telomere shortening, or mitochondrial dysfunction. While these processes occur commonly with age, their impact on cell fate and disease susceptibility are influenced by diverse regulatory factors. Additionally, many of the cellular responses to these stressors, including senescence, have homeostatic functions and are not universally pathologic. Therefore, nuanced therapeutic approaches will be required to target these processes. Such approaches may need to be cell- or tissue- specific or have modulatory rather than inhibitor effects on key pathways. Because of the fundamental regulatory role of non-coding RNAs, and the growing capacity for cell-specific targeting, non-coding RNAs may emerge as ideal therapies to target chronic lung disease and other age-related disorders.

## Author Contributions

NO and MS wrote the manuscript and built the tables, which were original. All authors have read and approved the submitted manuscript version.

## Conflict of Interest

The authors declare that the research was conducted in the absence of any commercial or financial relationships that could be construed as a potential conflict of interest.
